# Growth differentiation factor-15 and prognosis in acute respiratory distress syndrome: a retrospective cohort study

**DOI:** 10.1186/cc12737

**Published:** 2013-05-24

**Authors:** Brendan J Clark, Todd M Bull, Alexander B Benson, Amanda R Stream, Madison Macht, Jeanette Gaydos, Christina Meadows, Ellen L Burnham, Marc Moss

**Affiliations:** 1 Department of Medicine, Division of Pulmonary Sciences and Critical Care Medicine, University of Colorado Denver, 12700 East 19th Avenue, Aurora, CO 80045, USA

**Keywords:** Acute respiratory distress syndrome, pulmonary vascular dysfunction, risk prediction, growth differentiation factor-15

## Abstract

**Introduction:**

We sought to determine whether higher levels of the novel biomarker growth differentiation factor-15 (GDF-15) are associated with poor outcomes and the presence of pulmonary vascular dysfunction (PVD) in patients with acute respiratory distress syndrome (ARDS).

**Methods:**

We conducted a retrospective cohort study in patients enrolled in the Acute Respiratory Distress Syndrome Network Fluid and Catheter Treatment (FACT) Trial. Patients enrolled in the FACT Trial who received a pulmonary artery catheter (PAC), had plasma available from the same study day and sufficient hemodynamic data to determine the presence of PVD were included. Logistic regression was used to determine the association between GDF-15 level and 60-day mortality.

**Results:**

Of the 513 patients enrolled in the FACT Trial assigned to receive a PAC, 400 were included in this analysis. Mortality at 60 days was significantly higher in patients whose GDF-15 levels were in the third (28%) or fourth (49%) quartile when compared to patients with GDF-15 levels in the first quartile (12%) (*P *<0.001). Adjusting for severity of illness measured by APACHE III score, the odds of death for patients with GDF-15 levels in the fourth quartile when compared to the first quartile was 4.26 (95% CI 2.18, 10.92, *P *<0.001). When added to APACHE III alone for prediction of 60-day mortality, GDF-15 levels increased the area under the receiver operating characteristic curve from 0.72 to 0.77. At an optimal cutoff of 8,103 pg/mL, the sensitivity and specificity of GDF-15 for predicting 60-day mortality were 62% (95% CI 53%, 71%) and 76% (95% CI 71%, 81%), respectively. Levels of GDF-15 were not useful in identifying the presence of PVD, as defined by hemodynamic measurements obtained by a PAC.

**Conclusions:**

In patients with ARDS, higher levels of GDF-15 are significantly associated with poor outcome but not PVD.

## Introduction

Growth differentiation factor-15 (GDF-15) is a stress-responsive cytokine and member of the transforming growth factor-beta superfamily [1]. GDF-15 has been extensively studied as a biomarker for cardiovascular disease and was initially described as a marker of poor outcomes in acute coronary syndromes and chronic left-sided heart failure [2-8]. More recently, GDF-15 expression has shown promise as a biomarker for the identification of and prognosis in pulmonary vascular disorders including pulmonary embolism, idiopathic pulmonary arterial hypertension (PAH), and PAH associated with systemic sclerosis [9,10].

Acute respiratory distress syndrome (ARDS) is a devastating cause of respiratory failure that is commonly accompanied by pulmonary vascular and right ventricular dysfunction [11]. Despite promising data linking GDF-15 with poor outcomes in pulmonary vascular disorders, it has not been measured in patients with ARDS. The development of novel biomarkers may improve our understanding of ARDS by identifying more accurate and precise estimates of risk for poor outcomes in patients with ARDS and elucidating underlying mechanisms that drive these outcomes [12,13]. Biomarkers may also help identify subgroups of patients that respond differently to treatments. Studying therapies targeted to the subgroup of patients who develop pulmonary vascular dysfunction (PVD) in the setting of ARDS is difficult because pulmonary artery catheters are no longer part of the routine care in ARDS [14]. Therefore, a novel biomarker that is easy to measure could identify and target therapies to ARDS patients with PVD.

The Fluid and Catheter Treatment (FACT) Trial enrolled patients with ARDS and demonstrated that fluid management guided by a pulmonary artery catheter (PAC) does not lead to an improvement in clinical outcome when compared to fluid resuscitation guided by a central venous catheter [14,15]. The 501 patients who received a PAC as part of the study protocol now represent the largest available cohort of patients in which to study PVD in ARDS. Given the strong association of GDF-15 with poor outcomes in several diseases, we sought to determine the association between GDF-15 levels and mortality as well as measures of extrapulmonary organ failure in patients with ARDS. Furthermore, given the need for a noninvasive biomarker of PVD in patients with ARDS and the correlation of GDF-15 with elevated right ventricular systolic pressure in patients with scleroderma-associated pulmonary hypertension [10], we sought to determine the utility of GDF-15 levels in the identification of PVD in patients with ARDS enrolled in the FACT Trial. We hypothesized that higher GDF-15 levels would be associated with the presence of PVD and with poor outcomes including mortality and ventilator-free, ICU-free, and organ failure-free days.

## Methods

We conducted a secondary analysis of patients enrolled in the FACT Trial. Full inclusion and exclusion criteria for the study are available as part of the parent manuscript. Importantly, patients with severe chronic obstructive pulmonary disease or 'clinically significant pulmonary hypertension' were excluded [14]. Patients were included in this analysis if they were enrolled in the FACT Trial, randomized to the pulmonary artery catheter (PAC) treatment arm, and received a PAC. Patients were excluded if they did not have sufficient PAC measurements to calculate a pulmonary vascular resistance index (PVRi) and transpulmonary gradient (TPG) or did not have a plasma sample available.

### Measurement of GDF-15

GDF-15 was quantified in the day 1 plasma samples using the Human GDF-15 Quantikine ELISA Kit (R&D Systems, Minneapolis, MN, USA Catalog No. DGD150), following the manufacturer's protocol. All samples were obtained from the National Heart Lung and Blood Institute Biologic Specimen and Data Repository Information Coordinating Center (BioLINCC). Samples were initially diluted 4-fold with the Calibrator Diluent RD5-20 included in the kit, as recommended in the manufacturer's protocol. A subset of the plasma samples were out of the range of detection, and were diluted up to 40-fold with the Calibrator Diluent RD5-20 before requantification. GDF-15 values were adjusted to account for the dilution factor. ELISA plates were read on a VersaMax microplate reader (Molecular Devices; Sunnyvale, CA, USA), using SoftMax Pro v3.1.2 analysis software (MDS Analytical Technologies; Sunnyvale, CA, USA). Plates were read at 450nm, with wavelength correction of 540 nm. All assays were run in duplicate and results were averaged. In 92% of the patients the value of both measurements was within 15% of the average.

### Pulmonary vascular dysfunction

As previously described, we were unable to define PAH by the traditional criteria of a mean pulmonary artery pressure (mPAP) >25 mm Hg because 59% of the 500 patients with PACs in the FACT Trial had a pulmonary arterial occlusion pressure (PAOP) >15 mm Hg [11]. Therefore, we used the PVRi and TPG to define PVD. The PVRi is defined as: 80 × (mPAP-PAOP)/cardiac index. Normal values for the PVRi are 255 - 285 dynes - sec/cm^-5^/m^2^. We defined PVD as the presence of a PVRi >285 dynes - sec/cm^-5^/m^2 ^[16]. The TPG is calculated as: (PAP - mean PAOP) with PVD defined as a TPG >12 mm Hg. All hemodynamic measurements were obtained on study day 1 (the same study day that plasma samples were collected).

### Outcome variables

To analyze the association between GDF-15 levels and outcome, we chose 60-day mortality as the primary outcome variable. Secondary outcome variables included the number of extrapulmonary organ failure-free days, the number of ICU-free days, and the number of ventilator-free days. Organ failure-free days were calculated using the Brussels score, as previously described [17]. The Brussels score places renal, coagulation, cardiovascular, central nervous system, and hepatic failure into no organ dysfunction, mild, moderate, severe, or extreme organ dysfunction. Moderate, severe, or extreme organ dysfunction is considered to be clinically significant [17]. Patients were categorized into the presence or absence of clinically significant organ dysfunction for each study day. The number of days that the patient was alive without organ failure through study day 28 was then calculated as in prior ARDS Network studies [18-20]. To determine the association between GDF-15 levels and the presence of PVD, we used the presence of PVD as the outcome variable, as defined above.

### Statistical analysis

The Wilcoxon rank-sum test or Student's *t*-test were used to analyze differences in continuous variables between patients with and without PVD. Simple linear regression was used to test associations between continuous variables. The chi-square test was used to test for significant differences in categorical variables between groups. To test the association between GDF-15 levels and 60-day mortality, we initially performed logistic regression using log-GDF-15 level as the predictor and death at study day 60 as the outcome using the Hosmer-Lemeshow statistic to ensure goodness of fit. To ease interpretation, we then used quartiles of GDF-15 as the predictor of interest with the first quartile as the control group [4]. *A priori*, we included severity of illness measured by acute physiology and chronic health evaluation (APACHE) III score in our models because our goal was to test the ability of GDF-15 to predict outcomes. To test for an association between GDF-15 levels and organ failure-free days, we constructed separate multiple linear regression models using the log-GDF-15 quartile as the predictor and ICU-free days, ventilator-free days, and organ failure-free days as the outcome variable. *A priori*, we also included APACHE III score as a covariate in this model. We examined plots of residual versus predicted values to ensure that assumptions of linear regressions were met. Finally, to determine whether the association between GDF-15 levels and mortality is independent of other known predictors of poor outcomes in patients with ARDS, we performed a *post hoc *multiple logistic regression additionally adjusting for age, gender, race/ethnicity, baseline creatinine, and ARDS risk factor.

To further describe whether GDF-15 levels significantly add to APACHE III scores as a predictor of 60-day mortality, we also constructed receiver operating characteristic (ROC) curves with APACHE III alone and APACHE III plus GDF-15 level, then calculated the difference in the c-statistic. A c-statistic is commonly used to quantify the capacity of a biomarker of interest to discriminate between outcomes. Scores range from 0.5 to 1.0 with higher values indicating a better predictive model. For binary outcomes, the c-statistic is equivalent to the area under the ROC curve. A difference of 0.05 in the c-statistic with and without the biomarker of interest is generally considered to be clinically significant [21]. To examine the association between GDF-15 level and PVD, we constructed an ROC curve using the log-GDF-15 level as the predictor and the presence of PVD as the outcome.

### Protection of human subjects

The institutional review boards of each participating medical center or hospital approved the original FACT Trial and patients or surrogates provided informed consent prior to enrollment. The study was conducted in accordance with the established ethical standards of the medical centers and with the principles of the Declaration of Helsinki. The ARDS Network provided de-identified data for our analysis. Additional approval for this study was obtained from the Colorado Multiple Institutional Review Board (COMIRB Protocol No 10-0825).

## Results

**Figure 1:**
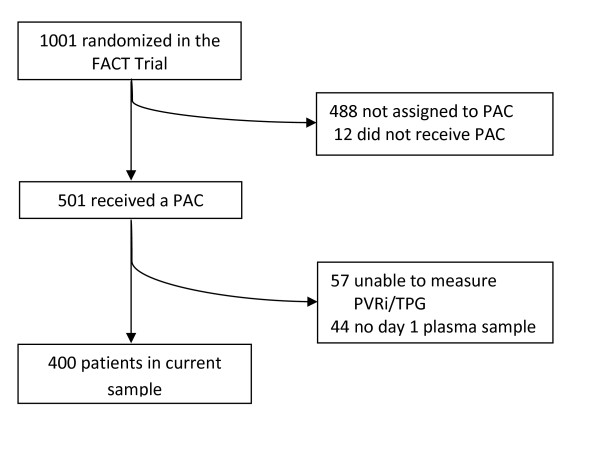
**Selection of patients for inclusion in this analysis**.

Of the 513 patients enrolled in the FACT Trial assigned to the PAC arm, 501 received a PA catheter. Of those that received a PAC, 400 patients were eligible for inclusion in this analysis (Figure 1). The most common reason that patients were excluded was the lack of an available day 1 measurement of PVRi or TPG (*n *= 57). There was no significant difference in age, 60-day mortality, severity of illness, or ventilator-free days between the patients who were excluded and the patients who were included in this study. PVD was present in 187 (47%) of the 400 patients when assessed using PVRi and 236 (59%) of the 400 patients when assessed using TPG. The overall 60-day mortality in the cohort was 26.5%. On average, patients who died were older, more likely to have sepsis as an ARDS risk factor, be immunosuppressed, and have higher APACHE III scores, baseline creatinine, and a lower PaO2/FiO2 ratio (Table 1). The median plasma GDF-15 level was 6,167 pg/mL (interquartile range (IQR) 3,585 pg/mL, 10,150 pg/mL) with a range of 880 pg/mL to 23,830 pg/mL. Only three of the 400 patients (0.7%) had GDF-15 levels under 1,200 pg/mL, the commonly accepted cutoff for normal values [4].

**Table 1:** Baseline characteristics of patients included in this analysis.

	Alive at 60 days (*n *= 294)	Dead at 60 days (*n *= 106)	*P *value
**Age**	47 [37,57]	51 [40,65]	0.01

**Gender **(% male)	51	57	0.35

**Race **(%)			0.04

White	70	55	

African American	20	30	

Hispanic	7	9	

Other	3	6	

**ARDS risk factor **(%)			

Sepsis	38	53	0.01

Aspiration	22	18	0.33

Pneumonia	64	67	0.62

Trauma	10	5	0.10

Other	9	6	0.30

**Comorbidities **(%)			

Diabetes mellitus	18	16	0.27

Cirrhosis	4	3	0.62

Immunosuppression	6	17	<0.01

**APACHE III score**	86 [67,106]	111 [91,132]	<0.01

**Creatinine **(mg/dL)	0.9 [0.7, 1.4]	1.3 [0.9, 1.9]	<0.01

**PaO2/FiO2**	153 [104, 210]	143 [94, 198]	0.17

**GDF-15 Level **(pg/mL)	5,222 [3,174, 12,748}	9,743 [5,811, 13,462]	< 0.001

Continuous variables are presented as median [interquartile range].

### GDF-15 level and mortality

**Figure 2:**
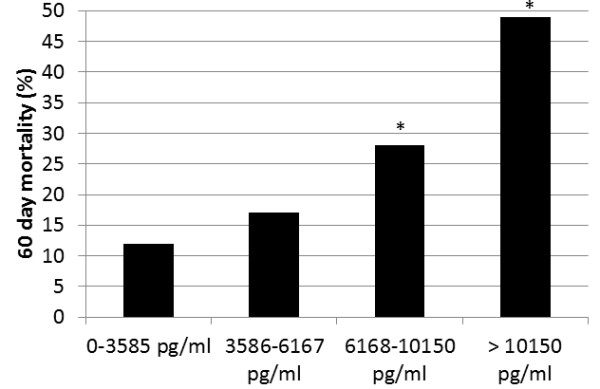
**Higher levels of growth differentiation factor-15 are associated with a higher 60-day mortality**. *P <0.001 when compared to quartile 1. *n *= 100 in each quartile.

**Figure 3:**
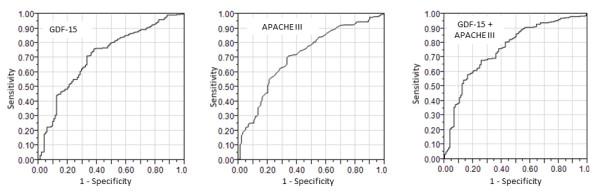
**Receiver operating characteristic curves for the prediction of 60-day mortality**.

Patients whose GDF-15 level was in the third or fourth quartile had a significantly higher 60-day mortality rate when compared to patients whose GDF-15 level was in the first quartile (Figure 2). The c-statistic for log-GDF-15 alone as a predictor of mortality was 0.72 while the c-statistic for APACHE III alone as a predictor of mortality was also 0.72. At an optimal cutoff of 8,103 pg/mL, the sensitivity and specificity of GDF-15 for predicting 60-day mortality were 62% (95% CI 53%, 71%) and 76% (95% CI 71%, 81%), respectively. When log-GDF-15 levels were added to APACHE III score as a predictor of 60-day mortality, the c-statistic for the ROC curve increased from 0.72 to 0.77 (Figure 3). Adjusting for severity of illness measured by APACHE III score, the odds of death at 60 days increased 2.43 times per log increase in GDF-15 (95% CI 1.65, 3.64; *P *<0.001). After adjusting for severity of illness measured by APACHE III score, patients whose GDF-15 level was in the highest quartile had a significantly higher odds of death when compared to patients whose GDF-15 level was in the first quartile (Table 2).

**Table 2:** Adjusted odds ratios for GDF-15 quartile as a predictor of 60-day mortality.

	Adjusted OR*	95% CI	*P *value
**1^st ^quartile**	Reference	--	--

**2^nd ^quartile**	1.20	0.53, 2.78	0.67

**3^rd ^quartile**	2.02	0.95, 4.52	0.07

**4^th ^quartile**	4.28	2.05, 9.42	<0.001

*Adjusted for severity of illness measured by acute physiology and chronic health evaluation (APACHE) III score.

In a multiple logistic regression model additionally adjusted for age, gender, race/ethnicity, baseline serum creatinine, and ARDS risk factor, the odds of death were increased 2.86 times per log increase in GDF-15 (95% CI 1.84, 4.54; *P *<0.001). In a multiple logistic regression model adjusting for the same covariates, the association between GDF-15 in the second and third quartiles were unchanged while the odds of death for patients in the fourth quartile of GDF-15 was 5.26 times that of patients in the first quartile (95% CI 2.31, 12.67; *P *<0.001).

### GDF-15 level and secondary outcomes

In a univariate analysis, patients with GDF-15 levels in the fourth quartile had significantly few ventilator-free and ICU-free days when compared to patients with GDF-15 levels in the first quartile (Table 3). Similarly, patients with GDF-15 levels in the fourth quartile had fewer cardiovascular, central nervous system (CNS), coagulation, renal, and hepatic failure-free days when compared to patients with GDF-15 levels in the first quartile. After adjusting for severity of illness measured by APACHE III scores, patients with GDF-15 levels in the third and fourth quartile had significantly fewer ICU-free and ventilator-free days when compared to patients with GDF-15 levels in the first quartile (Table 4). After adjusting for severity of illness, patients whose GDF-15 levels were in the third and fourth quartiles had significantly fewer cardiovascular, CNS, coagulation, renal, and hepatic failure-free days when compared to patients in the first quartile (Table 4).

**Table 3:** Ventilator-free, ICU-free, and specific organ system failure-free days by GDF-15 quartile.

	Quartile 1	Quartile 2	Quartile 3	Quartile 4	*P *value
**Ventilator-free days**	22 [17,24]	19 [2,23]	12 0[23]	2 0[20]	<0.001

**ICU-free days**	20 [13,23]	17 [6,21]	10 0[20]	0 0[19]	<0.001

**Cardiovascular**	26 [21,28]	24 [18,27]	24 [14,26]	19 [1,26]	<0.001

**Central nervous system**	28 [21,28]	21 [21,28]	21 0[28]	8 0[21]	<0.001

**Coagulation**	28 [28,28]	28 [24,28]	28 [14,28]	20 [1,28]	<0.001

**Renal**	28 [28,28]	28 [27,28]	28 [2,27]	14 [2,27]	<0.001

**Hepatic**	28 [28,28]	28 [25,28]	28 [18,28]	15 [2,28]	<0.001

Data are presented as median [interquartile range].

**Table 4:** Results of separate multiple linear regression models with ventilator-free days, ICU-free days, and specific organ failure-free days as the outcome variables and GDF-15 quartile as the predictor variable.

	Quartile 2			Quartile 3			Quartile 4		
	β*	**95% CI**	***P *value**	β *	**95% CI**	***P *value**	β *	**95% CI**	***P *value**
**Ventilator-free days**	-2.91	-5.61, -0.21	0.03	-5.27	-8.01, -2.53	<0.01	-6.56	-9.38, -3.74	<0.01
**ICU-free days**	-1.88	-4.39, 0.62	0.14	-5.25	-7.81, -2.71	<0.01	-6.25	-8.87, -3.63	<0.01
**Organ failure-free days**									
**Cardiovascular**	-0.85	-3.32, 1.61	0.50	-2.38	-4.87, 0.12	0.06	-6.03	-8.61, -3.45	<0.01
**CNS**	-2.03	-4.78, 0.72	0.15	-5.66	-8.44, -2.88	<0.01	-8.69	-11.56, -5.81	<0.01
**Coagulation**	-0.19	-2.60, 2.22	0.88	-3.03	-5.46, -0.59	0.02	-7.49	-10.01, -4.97	<0.01
**Renal**	-0.45	-2.87, 1.98	0.72	-4.73	-7.18, -2.27	<0.01	-10.0	-12.56, -7.49	<0.01
**Hepatic**	-0.01	-2.42, 2.41	0.99	-2.52	-4.96, -0.07	0.04	-8.49	-11.02, -5.97	< 0.01

Patients in GDF-15 quartile 1 served as the control group and, therefore, are not included in the table. *Parameter estimates shown are the difference when compared to patients in GDF-15 quartile 1 after adjusting for APACHE III score. CNS, central nervous system; 95% CI, 95% confidence interval.

### GDF-15 level as a predictor of pulmonary vascular dysfunction

**Figure 4:**
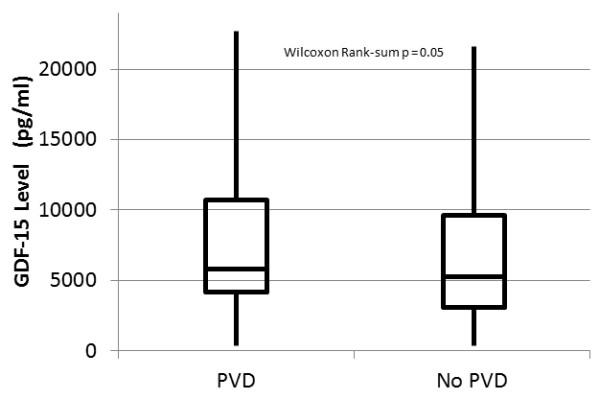
**The median (horizontal lines), interquartile range (box), and range (vertical lines) of GDF-15 levels in patients with and without pulmonary vascular dysfunction (PVD)**.

On average, for each log increase in GDF-15, PVRi increased by 41.2 dynes - sec/cm^-5^/m^2 ^(95% CI 17.3, 66.2 dynes - sec/cm^-5^/m^2^; r = 0.17; *P *<0.001). Similarly, for each log increase in GDF-15, TPG increased by an average of 1.33 mm Hg (95% CI 0.45, 2.20 mm Hg; r = 0.15; *P *= 0.003). However, there was no significant difference in the median level of GDF-15 in patients with PVD when compared to patients without PVD (*P *= 0.05, Figure 4). In a multiple logistic regression model adjusting for age, PaO2 to FiO2 ratio, creatinine, and APACHE III score, there was no significant association between the level of GDF-15 and the presence of PVD (adjusted OR 1.21 per log increase in GDF-15; 95% CI 0.86, 1.70; *P *= 0.27). A ROC curve with log-GDF-15 level as the predictor and the presence of PVD as the outcome variable yielded a c-statistic of 0.56. At an optimal cutoff of 5,105 ng/dL, GDF-15 had a sensitivity of 65% (95% CI 58%, 72%) and a specificity of 46% (95% CI 40%, 53%) for the identification of PVD.

## Discussion

In this large cohort of patients with invasive hemodynamic measurements used to define the presence of PVD, we demonstrated a dose-dependent and significant association between plasma GDF-15 levels and mortality in addition to several secondary outcomes in patients with ARDS. Of note, only three (0.7%) patients in this study had a GDF-15 level that was less than 1,200 ng/L, the commonly accepted cutoff for normal. In contrast, we demonstrated that, although plasma GDF-15 levels are associated with higher measurements of TPG and PVRi, there is significant variability in GDF-15 levels between patients with and without PVD. Thus, the sensitivity and specificity of GDF-15 as a single test for the identification of PVD are poor and plasma GDF-15 should not be used as a single biomarker for the identification of PVD in this population. We highlight that we chose to use TPG and PVRi to define PVD so that patients with an elevated PAOP would not be classified as having PVD.

Although many of the effects of GDF-15 remain unknown, recent work has begun to elucidate its role in acute coronary syndromes. In a series of experiments following coronary artery ligation in mice, Kempf and colleagues demonstrate that GDF-15 inhibits polymorphonuclear leukocyte recruitment by interfering with chemokine signaling and activation of integrins [8]. Because PMN recruitment and activation play a vital role in ARDS, it is possible that higher GDF-15 levels are expressed in an effort to attenuate this response [22]. Other biomarkers that are associated with poor outcomes have mechanisms that should be beneficial. For example, brain natriuretic peptide (BNP) has a sympathoinhibitory effect in patients with congestive heart failure [23]. However, higher plasma levels of BNP are a poor prognostic indicator in congestive heart failure [24-27]. A limitation of our study is that we did not measure proinflammatory cytokines. In the absence of this information, and without a more definitive understanding of the effects of GDF-15, it is not clear what mediates the association between GDF-15 and poor outcomes. GDF-15 may impair neutrophil recruitment and chemokine signaling thus leading to deleterious effects in ARDS. However, future basic, translational or clinical work would be necessary to determine whether GDF-15 mediates poor outcomes in ARDS.

In prior studies, several biomarkers have been associated with mortality in patients with ARDS including surfactant protein D [28], interleukin-8 [29], soluble tumor necrosis factor receptor-1 [30], von Willebrand factor [31], and soluble intracellular adhesion molecule-1 [32]. When combined into a panel, these biomarkers significantly improve risk prediction by APACHE III alone, increasing the area under the curve (AUC) from 0.680 to 0.747 in the derivation cohort and from 0.767 to 0.793 in a validation cohort [12]. It is of interest that GDF-15 alone increased the AUC by 0.05 in our study. However, it is not clear how GDF-15 would perform in a validation cohort. Furthermore, we cannot draw any conclusions regarding the performance of GDF-15 relative to other prognostic biomarkers in ARDS.

As highlighted in discussions by Ware, Calfee and colleagues [12,13], there are at least two important reasons to refine the ability to provide a precise prognosis for patients with ARDS. First, in large randomized controlled trials, the mortality in control groups has declined over time. In the three most recent ARDS network studies, mortality in the control group ranged from 16 to 23% [33-35]. While this may be due to important advancements in the care of patients with ARDS, it creates significant logistical challenges for the conduct of future treatment trials. Specifically, absent treatments that demonstrate a dramatic improvement in mortality, randomized controlled trials that have sufficient power to demonstrate a difference in outcomes may be cost prohibitive. Therefore, the ability to select a population with a higher mortality but who may still benefit from treatment may become more imperative. The ability to do this with the greatest precision offers the opportunity to advance clinical research in ARDS through the cost-efficient conduct of treatment trials.

Second, future therapies for ARDS may have significant undesired side effects that place patients at additional risk for poor outcomes. In that case, the ability to develop an accurate and precise estimate of mortality for a given patient may help weigh the risks and benefits of a treatment and, therefore, identify patients most likely to derive an overall benefit. Such an approach has been used to treat acute coronary syndromes in the form of the Thrombolysis in Myocardial Infarction (TIMI) risk score. Of note, GDF-15 may provide prognostic information above and beyond the information provided by the TIMI risk score highlighting its ability to independently improve mortality prediction for other patient populations [2].

There are important limitations to this study. First, although the plasma samples were obtained on the same study day as the invasive hemodynamic measurements used to calculate the PVRi, the half-life of GDF-15 is only 20 minutes. The timing of plasma collection relative to hemodynamic measurements was not collected in the FACT Trial. Therefore, the utility of GDF-15 as a biomarker for the identification of PVD in patients with ARDS may have been obscured in our study. Furthermore, because GDF-15 measurements were obtained at a single point in time, we cannot make inferences regarding GDF-15 levels later in the course of disease. For example, persistently elevated GDF-15 levels may be more strongly correlated with poor outcomes. Second, we did not validate GDF-15 as a predictor of outcomes in ARDS in a separate cohort. This would be necessary before drawing any conclusions regarding the clinical utility of GDF-15 as a predictor of outcomes in patients with ARDS. Third, the patients in this cohort were enrolled in a randomized controlled trial. Thus, there could be a selection bias that could limit the ability to generalize our findings to patients with ARDS in general. Finally, nearly all of the patients included in this analysis had moderate ARDS by Berlin criteria [36]. Therefore, our findings may not apply to patients with mild or severe ARDS.

## Conclusion

In patients with ARDS enrolled in the FACT Trial who received a PAC, we demonstrated that higher plasma GDF-15 levels are associated with an increased mortality and fewer ventilator-free, ICU-free, and organ failure-free days. Further studies are needed to understand how GDF-15 performs as a risk predictor in a separate cohort and to understand how GDF-15 performs relative to other known biomarkers for poor outcomes in patients with ARDS.

## Key messages

• Higher levels of growth differentiation factor-15 are associated with poor outcomes in patients with ARDS including a higher odds of death at 60 days, fewer ICU-free and ventilator-free days, and fewer organ failure-free days.

• Although GDF-15 levels are associated with transpulmonary gradient and pulmonary vascular resistance index, GDF-15 was not useful as a single biomarker for the identification of PVD in patients with ARDS.

• The timing of GDF-15 measurements relative to hemodynamic measurements in this study is not known. Thus, GDF-15 levels may be better at identifying the presence of PVD than we report.

• Future studies are needed to validate the usefulness of GDF-15 as a prognostic biomarker and to compare its utility relative to other known biomarkers in patients with ARDS.

## Abbreviations

AUC: area under the curve; APACHE: acute physiology and chronic health evaluation; ARDS: acute respiratory distress syndrome; BNP: brain natriuretic peptide; CNS: central nervous system; ELISA: enzyme-linked immunosorbent assay; FACT: Fluid and Catheter Treatment; GDF-15: growth differentiation factor-15; IQR: interquartile range; mPAP: mean pulmonary arterial pressure; PAC: pulmonary artery catheter; PAH: pulmonary arterial hypertension; PAOP: pulmonary artery occlusion pressure; PVD: pulmonary vascular dysfunction; PVRi: pulmonary vascular resistance index; ROC: receiver operating characteristic; TIMI: thrombolysis in myocardial infarction; TPG: transpulmonary gradient.

## Competing interests

TMB, ELB, and MM have received grant funding from the National Heart Lung, and Blood Institute. TMB received an investigator-initiated grant from United Therapeutics. TMB serves on the advisory board for Actelion.

## Authors' contributions

BJC helped conceive and design the study, acquire, analyze, and interpret the data, and drafted and revised the manuscript. TMB helped conceive and design the study, acquire, analyze, and interpret the data, and revise the manuscript. ABB and MMa helped with interpretation of the data and revision of the manuscript. ARS helped with interpretation of the data and revision of the manuscript. JG and CM performed all GDF-15 measurements and helped draft the manuscript. ELB helped with interpretation of the data and manuscript revision. MM helped with the conception and design of the study, data analysis and interpretation, and manuscript revision. All authors read and approved the final manuscript.
